# DEVELOPMENT AND ASSESSMENT OF THE PERFORMANCE OF A LARGE LANGUAGE MODEL FOR ADMINISTERING THE SHORT BLESSED TEST

**DOI:** 10.1093/geroni/igae098.3948

**Published:** 2024-12-31

**Authors:** Rafeeul Jaman, Sarah Nessen, Michael Adjei-Poku, Joella Byerley, Olivia Sailors, Jason Karlawish, Kyra O’Brien, Ari Friedman

**Affiliations:** University of Pennsylvania, Lansdoqne, Pennsylvania, United States; University of Pennsylvania, Philadelphia, Pennsylvania, United States; University of Pennsylvania, Philadelphia, Pennsylvania, United States; University of Pennsylvania, Philadelphia, Pennsylvania, United States; University of Pennsylvania, Philadelphia, Pennsylvania, United States; University of Pennsylvania, Philadelphia, Pennsylvania, United States; University of Pennsylvania, Philadelphia, Pennsylvania, United States; University of Pennsylvania, Philadelphia, Pennsylvania, United States

## Abstract

Universal health system-based screening for mild cognitive impairment (MCI) or impairment consistent with dementia can modify care and increase access to disease-modifying therapies and memory centers, potentially reducing disparities. Constraints on nurse and clinician time have hindered existing efforts. Large language models (LLMs) present a potential solution. However, they are subject to confabulation and poor arithmetic accuracy, and were not trained on data containing definitive assessments of cognitive impairment. We hypothesized that using an LLM to administer the Short Blessed Test (SBT) and calculating test scores from item scores would mitigate these errors. We conducted prompt engineering on OpenAI GPT4o to construct an interactive tool. A team member then simulated being patients with different levels of cognitive impairment across an orthogonal matrix spanning the 6 SBT item scores, answering each question to achieve the intended item score. We summed the LLM-reported item scores to the total score that should have been obtained based on the input answers. We evaluated 57 sets of item scores with total scores of 2-26. Of these, 54.7% had an LLM score equal to the true score. All errors except 2 were within 3 points of the true score. Using a cutoff of >=5 points for mild cognitive impairment, specificity was 100.0%, and negative predictive value was 50%. LLMs may play a role in reducing nurse and clinician screening burden in hospital settings, where the benefit of being able to conduct screening at all outweighs the possibility of missed diagnoses, but requires human confirmation.

